# Malaria: How Are We Doing and How Can We Do Better?

**DOI:** 10.4269/ajtmh.18-0997

**Published:** 2019-01-08

**Authors:** Philip J. Rosenthal, Chandy C. John, N. Regina Rabinovich

**Affiliations:** 1 Department of Medicine, University of California, San Francisco, San Francisco, California;; 2 Department of Pediatrics, Indiana University, Indianapolis, Indiana;; 3 Harvard T.H. Chan School of Public Health, Boston, Massachusetts;; 4 ISIGlobal, Boston, Massachusetts

The annual World Malaria Report from the WHO provides the latest update on progress against malaria.^1^ By combining data—where they exist—and modeling—where data are absent—the report offers the malaria community a valuable annual update. How many cases and deaths from malaria are estimated for the prior year, and what are we doing to make things better? Gathering the relevant numbers is challenging, as the great majority of malaria cases are not reported. Counting deaths and sorting out their causes in malaria-endemic regions is inexact. Nonetheless, the report offers carefully determined estimates, and it provides a valuable overview, with dozens of illustrative figures, tables, and graphs. Looking at the 2018 report, how are we doing?

Malaria control has a storied past. Fueled by the availability of effective treatment with chloroquine and vector control with DDT, the WHO Global Malaria Eradication Program was launched in 1955. The program contributed to some dramatic successes, including elimination of malaria in the United States and Europe, and dramatic decreases in parts of Asia and South America (a decrease to 17 reported cases in Sri Lanka in 1963).^2^ But, elimination was not seriously attempted in Africa because of perceived logistical challenges, known very high transmission intensity, and disappointing results of a pilot project in Nigeria (the Garki Project).^3^ Moreover, sustaining the gains where elimination was not achieved was challenging (e.g. resurgence to hundreds of thousands of cases annually in Sri Lanka by the late 1960s).^2^ The Global Malaria Eradication Program was put on hold by the WHO in 1969, with focus turned to malaria control. The following years were marked by fragmentation of public health programs to deliver insecticides for prevention and drugs for treatment, and the recognition of increasing resistance of mosquitoes to DDT and of malaria parasites to chloroquine. By the 1990s, the annual death toll from malaria was likely the greatest in human history.

The tide began to turn about 20 years ago, with a consensus that we needed to do better; increased funding from many agencies for malaria research and control; and establishment of new programs, including the Global Fund for AIDS, Tuberculosis, and Malaria (the Global Fund) and the United States President’s Malaria Initiative. With increased political commitment and investment came the ability to best utilize the new toolbox for malaria control, notably artemisinin-based combination therapies (ACTs), rapid diagnostic tests, and long-lasting insecticidal nets. Endemic countries were empowered and committed to “rolling back malaria.” In parallel, there was a concerted effort to develop better tools, with the creation of the Malaria Vaccine Initiative, the Medicines for Malaria Venture, the Foundation for Innovative New Diagnostics, and the Innovative Vector Control Consortium, as well as increased investment in malaria research by the United States National Institutes of Health and other national funding bodies. In 2007, at a well-publicized meeting in Seattle, Bill & Melinda Gates implored the public health community to raise their sights and not be satisfied with only the control of malaria. Rather, they challenged leaders to revisit the long-term goal of malaria eradication. The community slowly warmed to this risky, yet exciting concept. Just over a decade later, how are we doing?

Annual World Malaria Reports have had promising stories to tell over the last two decades. The numbers have generally been getting better year by year. Elimination is increasingly within reach. As noted in the most recent report, of 87 endemic countries, there are 47 with less than 10,000 reported cases and 24 with less than 100; these are at the frontline of the elimination effort. Sri Lanka, Paraguay, and Uzbekistan were recently certified as malaria free, with documented interruption of transmission for 3 years. There are 21 countries with a goal to eliminate malaria by 2020, and about half of them are likely to achieve this goal. Remarkably, in 2017 no indigenous cases of malaria were reported in China, and malaria in that country is now primarily a problem of returned travelers. So, we have seen great successes, but have these been shared around the globe?

The biggest challenge remains sub-Saharan Africa, the region responsible for about 90% of the worldwide malaria burden. Success has been seen in parts of Africa, for example much of southern Africa, Senegal, and Zanzibar. The 2018 report, however, documents a concerning reversal of gains, with increases in the number of malaria cases in 2017 in all of the 10 African countries with the highest malaria burdens, and an estimated 3.5 million more cases in these countries than in 2016. On the other hand, deaths from malaria are estimated to have continued to decrease year by year in Africa and worldwide. Overall, we are unlikely to meet the WHO Global Technical Strategy for Malaria milestones for 2020—to reduce both the incidence of malaria and malaria death rates by 40% compared with levels in 2015. The cover of the 2017 World Malaria Report displayed a signpost—we had reached a crossroad. The cover of the 2018 World Malaria Report shows converging train tracks—does this represent getting back on track, with convergence of opinions on how best to manage malaria, or an impending collision? What is the path forward?

Efforts to eliminate malaria in the countries that are now close will continue. However, there is consensus that we need to pay more attention to those countries that have persistent enormous burdens. The WHO has responded to this situation with a new plan, “high burden to high impact,” which emphasizes improved malaria control in the 10 countries in Africa with the greatest malaria burdens (Burkina Faso, Cameroon, Democratic Republic of the Congo, Ghana, Mali, Mozambique, Niger, Nigeria, Tanzania, and Uganda) plus India, together responsible for about 70% of the world’s malaria burden. The key elements of the new program are increased political will to decrease malaria deaths, strategic use of information to drive impact, use of best evidence by the WHO to provide global guidance, and coordinated national responses.

Efforts to control and eventually eradicate malaria will benefit from new tools to overcome the challenges we are currently facing. Vector control is challenged by resistance to pyrethroid and other insecticides. New bednets containing combinations of a pyrethroid plus the synergist piperonyl butoxide,^4^ or plus the insect growth regulator pyriproxyfen,^5^ offer improved preventive efficacy compared with traditional pyrethroid nets. However, these products and newer non-pyrethroid insecticides for use in bednets or indoor residual spraying add to the expense of mosquito control programs. New insecticides are also on the horizon.^6^ Further away, introduction of genetically modified mosquitoes incapable of transmitting malaria may contribute to malaria control.^7^

Use of ACTs to treat malaria has likely played a key role in decreasing malaria deaths, but ACT efficacy is seriously challenged in parts of Southeast Asia because of resistance to artemisinins and partner drugs.^8^ However, efficacy of ACTs appears to remain strong in most other areas, in particular the high-burden regions of Africa.^9^ Increased utilization of drugs to prevent malaria has promise in Africa, including sulfadoxine-pyrimethamine, which is simple to administer, but limited by drug resistance, as intermittent preventive therapy (IPT) in pregnancy; amodiaquine plus sulfadoxine-pyrimethamine as seasonal malaria chemoprevention in children in areas, mostly in West Africa, with highly seasonal transmission and relatively little drug resistance^10^; dihydroartemisinin-piperaquine, which has shown excellent preventive efficacy as IPT in children^11^ and pregnant women^12,13^ and as mass drug administration^14^; and azithromycin, which has modest antimalarial efficacy^15^ and, remarkably, has led to improved survival in African children.^16^ Chemoprevention is particularly relevant in high-burden countries, but elimination efforts can also use focused strategies to eliminate hotspots, including mass drug administration and targeted administration of drugs to contacts of identified patients or other high-risk individuals.^17^ A robust pipeline of malaria drugs is under development; with resistance likely growing over time, this pipeline offers critical new agents for treatment and prevention.^18^

A highly effective vaccine has been a leading priority for many years, but development has been challenging. The RTS,S vaccine, which targets invasive sporozoites, has consistently demonstrated modest preventive efficacy in African children,^19^ and is now under evaluation in a large, three-country implementation study. Other vaccines incorporating attenuated sporozoites and various parasite antigens are under intensive study.^20,21^ Regular seasonal immunization may overcome the loss of efficacy of some vaccines over time.^22^

An additional challenge is how to integrate the best available tools into country systems and to target, phase, and combine their use for maximum impact. Implementation science research can help us learn how to best integrate malaria management into primary care, avoid potential adverse consequences of a solely vertical focus on malaria, and offer early and appropriate evaluation and management of children with severe malaria (e.g. rectal artemisinins followed by rapid transport to referral centers^23^). With research advances, it is critical that these are translated into best practices within national public health policies.

Increased efforts to control and eliminate malaria will require increased funding. Funding increased dramatically early in the century, although it never reached more than 50% of projected needs. More recently, over about the last 5 years, funding has been flat.^1^ Proposed approaches to secure the needed support include long-term financing through the Global Fund; funding from governmental bilateral programs, including the United States President’s Malaria Initiative and the United Kingdom Department for International Development; and, most importantly, increased investment from malaria-endemic countries.

In summary, the 2018 World Malaria report tells us that our glass is half-full (malaria is eliminated or nearing that state in dozens of recently endemic countries; deaths are decreasing) and half-empty (malaria incidence has worsened in all of the 10 highest-burden countries in Africa). We will not reach the most rosy predictions from a decade ago, but on the other hand, we can celebrate some spectacular accomplishments in malaria control and elimination, especially outside of Africa. In Africa, although it has been increasingly difficult to bring down the disease burden, the number of malaria deaths continues to decrease despite rapid population growth. For both countries nearing elimination and high burden countries seeking improved control, the blueprint for success is quite clear.

First, we need to better understand the situation. The World Malaria Report estimates have been very helpful, but these estimates differ from those from other sources that use different modeling methodologies.^24^ Continued work to optimize methodologies and provide the most accurate estimates of the burden of malaria is needed. Potential tools to improve assessments include regular intensive data collection at representative surveillance sites; improved modeling, potentially incorporating new tools such as cell phone data or drone-based ecological surveys; and improved national reporting systems utilizing electronic data capture and rapid transmission to policymakers.

Second, a range of approaches addressing both elimination in countries nearing that goal and control in high-burden countries must be strongly supported. Successful efforts to achieve elimination should be celebrated, but successes only in low-burden countries will be difficult to justify if they are not complemented by equally successful efforts to reduce malaria incidence and mortality in high-transmission areas.

Third, we need to better utilize the excellent tools already available, and additional tools as they become available, to maximize progress for both elimination and control. Research to continue development of innovative new tools must be emphasized, with increased funding for research on vector control, drugs for treatment and prevention, vaccines, diagnostics, and implementation science.

Fourth, we need to make sure that what is learned from improved surveillance and new research impacts policy. Implementation science research toward improving our ability to respond to changing data and integrate research findings into public health policy is critical.

Fifth, substantially more financial and logistical support for malaria control and prevention will be required to do the needed work. This support will require increased investment from donor agencies and funders and, importantly, from the low- and middle-income countries that bear the overwhelming majority of the world’s malaria burden. We must guard against a “zero sum game” pitting elimination and control agendas against each other. Rather, as endorsed by the WHO, we must prioritize both elimination, where it is achievable, and improved control in the many countries where the malaria burden remains very high.
